# Cognitive and emotional demands of black humour processing: the role of intelligence, aggressiveness and mood

**DOI:** 10.1007/s10339-016-0789-y

**Published:** 2017-01-18

**Authors:** Ulrike Willinger, Andreas Hergovich, Michaela Schmoeger, Matthias Deckert, Susanne Stoettner, Iris Bunda, Andrea Witting, Melanie Seidler, Reinhilde Moser, Stefanie Kacena, David Jaeckle, Benjamin Loader, Christian Mueller, Eduard Auff

**Affiliations:** 1 Department of Neurology, Medical University of Vienna, Waehringer Guertel 18-20, 1090 Vienna, Austria; 2 Department of Otorhinolaryngology, Medical University of Vienna, Vienna, Austria; 3 Faculty of Psychology, University of Vienna, Vienna, Austria

**Keywords:** Black humour processing, Verbal intelligence and nonverbal intelligence, Mood disturbance, Aggression, Frame-shifting, Blending

## Abstract

Humour processing is a complex information-processing task that is dependent on cognitive and emotional aspects which presumably influence frame-shifting and conceptual blending, mental operations that underlie humour processing. The aim of the current study was to find distinctive groups of subjects with respect to black humour processing, intellectual capacities, mood disturbance and aggressiveness. A total of 156 adults rated black humour cartoons and conducted measurements of verbal and nonverbal intelligence, mood disturbance and aggressiveness. Cluster analysis yields three groups comprising following properties: (1) moderate black humour preference and moderate comprehension; average nonverbal and verbal intelligence; low mood disturbance and moderate aggressiveness; (2) low black humour preference and moderate comprehension; average nonverbal and verbal intelligence, high mood disturbance and high aggressiveness; and (3) high black humour preference and high comprehension; high nonverbal and verbal intelligence; no mood disturbance and low aggressiveness. Age and gender do not differ significantly, differences in education level can be found. Black humour preference and comprehension are positively associated with higher verbal and nonverbal intelligence as well as higher levels of education. Emotional instability and higher aggressiveness apparently lead to decreased levels of pleasure when dealing with black humour. These results support the hypothesis that humour processing involves cognitive as well as affective components and suggest that these variables influence the execution of frame-shifting and conceptual blending in the course of humour processing.

## Introduction

### Definition of black humour

 Black humour is defined as a kind of humour that treats sinister subjects like death, disease, deformity, handicap or warfare with bitter amusement (Mindess et al. 1985; Baldick 2001) and presents such tragic, distressing or morbid topics in humorous terms (Oxford dictionaries 2016). Black humour, often called grotesque, morbid, gallows or sick humour (Mindess et al. 1985; Oxford dictionaries 2016), is used to express the absurdity, insensitivity, paradox and cruelty of the modern world. Characters or situations are usually exaggerated far beyond the limits of normal satire or irony, potentially requiring increased cognitive efforts to get the joke. Furthermore, black humour, often uses devices associated with tragedy, is sometimes equated with tragic farce (Lagasse et al. 2000) and is perceived as morbid, nasty, psychopathic, twisted and often very funny (Maxwell 2003).

### Models of humour processing

Humour often uses categories and structures of thought organized in the form of frames which are accessed by particular images, notions or mappings (Lakoff 1987). In the course of humour processing, these categories and structures of thought are often semantically reanalysed and reorganized by mapping elements of one frame into a new frame (Coulson and Kutas 1998). Such a ‘frame-shifting’ process (Coulson 2000) can often be seen as the basis of humour processing as it requires the recruitment and integration of background knowledge about the frames used in a joke. Reading the joke ‘I let my accountant do my taxes because it saves time. Last spring it saved me ten years!’, the first sentence provokes the image of a busy-professional paying an accountant to do his taxes as the reader recalls his knowledge about relationships between business people and their accountants. However, the word ‘years’ in the latter sentence calls forth a reinterpretation of the word ‘time’ as time in prison, evoking a shifting of the initial frame ‘busy-professional’ into the frame ‘crooked-businessman’ (Coulson and Williams 2005). Another cognitive operation that underlies humour processing is called ‘blending’ which requires people to combine cognitive models from different domains into new concepts (Coulson 2001). In such a way, humour is often based on ‘frame blends’ (Hofstadter and Gabora 1989) which require the blurring of two distant scenarios so as to create a humorous hybrid situation composed of aspects of each situation. Such a form of blending can be demonstrated by a black humour cartoon by Stein (1997), used in the current study (see Table 1, cartoon 12). The cartoon shows the suicide of a husband who hanged himself with a green tie and is found by his wife and her friend. Finding her husband dangling from the ceiling, the wife is supposed to have feelings like shock, sadness or desperation. However, the elements of the tragic suicide of the husband are blended with the elements of the stereotypical complaining of a wife about her husband’s taste for clothing (‘And once again the green tie with the blue suit. Come on, what have I been nagging him about for all these years?’). It was shown that these operations underlying humour processing depend on cognitive abilities (Coulson 2001) and that increased cognitive ability is necessary to understand a joke (Coulson and Kutas 1998).

**Table 1:** Verbal description of the black humour cartoons (Stein 1997)

Cartoon	Situation	Text
1.	Santa Claus, standing on a long, thin tail and having some drops of blood under and on both boots, has been giving Christmas presents to a penguin, a dog and a cat, standing in front of him. Having distributed a fish, a dog biscuit and a tuna tin to them, he still has a gift wrapped cheese left in his hands.	Santa Claus: ‘And who put the cheese on his letter to me?’
2.	Death, impersonated by a skeleton in a hooded coat holding an hourglass and a sickle stands at the doorstep of a man’s apartment.	The man: ‘I am sorry, we do not die at the front door.’
3.	Up on a veritable height a man stands at the outer windowsill of an apartment block. Having a noose laid around his neck and a fixed knife directed to his stomach he puts a gun against his head. Beside him on the sill lies an emptied bottle labelled as poison and an envelope. Inside the apartment are two police officers, one of them pointing at the man saying:	‘Hey – I know this guy from elementary school. I remember that we called him Eberhard, the efficient.’
4.	A man scratching his chin apparently out of confusion is clutching the receiver of a public phone box. The voice coming from the receiver says:	‘Here is the answering machine of the self-help association for Alzheimer patients. If you still remember your topic, please speak after the tone.’
5.	A general practitioner is explaining the result of a medical test to a couple with her being pregnant:	‘To begin with, here is the good news: Your child will always find a parking space.’
6.	Four men are standing high up on a bungee jumping platform. One of them is holding a rope fixed on the one end to the platform. The other end of the rope is tied around a leg prosthesis that is turned upside down. One of them is telling the others:	‘I didn’t examining his certificate of disability in all detail.’
7.	A group of surgeons in an operating theatre is in the middle of what looks like a heart surgery. Without a sign of warning the heart springs out of the patient’s body right into one of the surgeons’ faces. Another surgeon remarks:	‘That’s the most amazing case of tissue rejection I’ve ever seen!’
8.	In a morgue a physician is lifting a white cover sheet off a body with a woman standing beside him. The woman confirms:	‘Sure, that’s my husband – anyway, which washing powder did you use to get that so white?’
9.	Two women, apparently real chatterboxes, are having a chat over coffee.	The first one: ‘He is crippled, she is crippled and what’s more they are going to have a baby.’ The other one: ‘I do hope things straighten themselves out.’
10.	In an operating theatre a surgeon has one arm deep in an opened body. Another surgeon explains the situation to a man in a suit:	‘The autopsy is finished; he is only looking for his wrist watch.’
11.	A dentist is on a root canal job with the patient being completely tensed up due to pain. At the back of the patient’s chair the tip of a rotating dental drill, apparently having worked its way through the patient’s mouth and neck comes into sight. The dentist asks his patient:	‘Does it hurt?’
12.	After having committed suicide the body of a man hangs from a light fixture in a living room, hung by his tie. His wife enters the room with a friend and looking at him she complains:	‘And once again the green tie with the blue suit. Come on, what have I been nagging him about for all these years?’

### Cognitive demands in humour processing

Investigations on the effects of cognitive domains involved in humour comprehension are often based on the ‘incongruity-resolution model’ (Suls 1972) which assumes that humour is processed within a two-stage problem-solving process. In the light of this model, humour processing is dependent on the recall of necessary background knowledge from the long-term memory (Coulson 2000) as well as problem-solving ability (Suls 1972). Humour processing was shown to be dependent on intelligence (Vrticka et al. 2013) as well as on verbal and visual abilities (Shammi and Stuss 1999) as these cognitive abilities potentially influence frame-shifting and cognitive blending. Whilst Feingold and Mazzella (1991) found appreciable associations between verbal measures and humour reasoning, Wierzbicki and Young (1978) found that verbal intelligence was positively related to humour comprehension. In the context of the previously mentioned incongruity-resolution model, Greengross and Miller (2011) showed that humour ability was more strongly associated with verbal intelligence than with abstract reasoning. Therefore, humour processing is assumed to be a complex information-processing task, relying heavily on intellectual as well as other cognitive abilities (e.g. Derks et al. 1997; Greengross and Miller 2011; Shammi and Stuss 1999; Vrticka et al. 2013).

### Emotional demands in humour processing

The notion of humour processing involves cognitive as well as emotional aspects (Ruch and Ekman 2001) and is supported by recent fMRI studies (e.g. Vrticka et al. 2013; Wild et al. 2003). In this context, it could be shown that higher intelligence not only influences the cognitive aspects of humour processing but also the affective components (Vrticka et al. 2013).

Considering the role of mood in humour appreciation, Deaner and McConatha (1993) showed that increased depression scores are associated with greater problems in the use of humour to cope with stressful events. Neumann and colleagues (2001) showed that subjective humour response is influenced by pre-existing mood as pre-existing mood increases the intensity of affectively congruent emotions whilst dampening incongruent emotions. According to Ruch and Köhler (1998), trait cheerfulness, seriousness and bad mood are the temperamental basis of humour. Whilst individuals with a high level of trait cheerfulness usually present a low threshold for laughter, individuals with trait bad mood do not seem to be able to be involved in humour or show sad or ill-humoured behaviour in cheerfulness-evoking situations. On the other hand, Ruch and Köhler (1998) as well as Remplein (1956) suggest that trait bad mood might facilitate the appreciation of certain forms of humour as ‘…someone in a bad mood might be prone to negative humour, e.g. enjoy humour of misanthropic quality…’ (Ruch and Köhler 1998, p. 211).

Theories about the relationship between aggression and humour were postulated as early as 1905 as Freud hypothesized that humour allows for a temporarily and relatively safe release of usually repressed sexual and aggressive urges in the form of wits. Therefore, aggressive humour ‘has at its disposal sources of pleasure to which harmless wit has no access’ (Freud 1905, p. 138). In a rating task study, McCauley and colleagues (1983) found a substantial association between humour and aggression as independent groups of subjects judged a set of cartoons. Prerost (1983) showed that individuals in an aggressive mood perceived aggressive humour as funnier than subjects in a non-aggressive mood. Moreover, the arousal levels in individuals in an aggressive mood lead to an increased appreciation of aggressive humour.

Herzog and Bush (1994) investigated the preference for sick jokes in a sample of 302 undergraduate students. They showed that the most preferred jokes were rated lowest in vulgarity and at the same time highest in fit and surprise. In another study, Herzog and Karafa (1998) investigated the preference for sick jokes (categories classified by Herzog and Bush 1994: death, death-baby, general and handicapped) compared to non-sick jokes (categories classified by Mindess et al. 1985: nonsense, social satire, philosophical, sexual hostile, demeaning to men and women, ethnic and scatological jokes—humour that possibly but not necessarily taps serious subjects but not sinister and tragic subjects like sick humour does) in a sample of 241 undergraduate students. Results showed that sick jokes were less preferred than non-sick jokes, but at the same time sense of humour showed a strong positive association with preference for sick jokes. Whilst fit and surprise were positively related to preference, cruelty (‘How vicious or cruel is the emotional tone of the joke towards an individual or group?’) was negatively related to preference. Other studies about sick humour showed that subjects who prefer such humour are more likely to be male (Herzog and Anderson 2000; Herzog and Karafa 1998; Oppliger and Zillmann 1997), more likely to be rebellious (Oppliger and Zillmann 1997) and are more capable to treat sick humour as playful fiction (Mindess et al. 1985).

### Aim of the study

Humour processing is a complex information-processing task that is dependent on a number of cognitive as well as emotional demands. The aim of the current study was to identify groups which differ with respect to the processing of black humour as it apparently provides a perfect combination of both cognitive and emotional demands. It was assessed whether these groups show differences with respect to cognitive (verbal and nonverbal intelligence) as well as emotional demands (mood disturbance, aggression).

## Materials and methods

### Participants

A total of 156 individuals, 76 females (49%) and 80 males (51%), participated in the study [sample size was calculated using G*Power (Faul et al. 2007)] showing a mean age of 33.4 years (SD = 11.9). With respect to education, 15 (10%) subjects had left school after the compulsory nine-year school programme, 52 (33%) had graduated from a two-or three-year high school programme, 72 (46%) individuals had graduated from a four-or five-year high school programme, and 17 (11%) subjects held a university degree. Eighty-two individuals (53%) were single, 60 (39%) were married, 11 (7%) were divorced, and 3 (15%) were widows.

### Methods

#### Black humour

Black humour processing was assessed using 12 black humour cartoons from ‘The black book by Uli Stein’ (Stein 1997). The chosen cartoons (see a verbal description of the cartoons in Table 1) deal with themes of death (*n* = 6; 50%), disease (*n* = 2, 17%), physical handicap (*n* = 3, 25%) or medical treatment (*n* = 1, 8%). The cartoons were shown in the original coloured version. Following Herzog and Bush (1994), the participants rated the cartoons with respect to *difficulty* (‘How hard is it to understand the humour of this joke, to get the point?’), *fit* (‘How well does the punch line seem to fit the situation leading up to it?’), *vulgarity* (‘How vulgar or tasteless is this joke?’), *surprise* (‘How surprised are you by the punch line of this joke, how unexpected is it?’), *novelty* (‘How novel, new, fresh is this joke?’), *interest* (‘How interesting do you find the topic or subject matter of this joke?’) and *preference* (‘How much do you like the joke, for whatever reason?’). Subjects had to rate the cartoons with respect to these categories, using a 4-point Likert scale ranging from ‘not at all’ to ‘a great deal’ for each category. The 4-point Likert scale was used to avoid having a midpoint and in this way to force a choice.

#### Verbal and nonverbal intelligence

Verbal intelligence was assessed by the ‘Vocabulary test’ (Schmidt and Metzler 1992) which requires the recognition of an existing word that is presented simultaneously with five distractor words (e.g. ‘ronoly – unidase – orisal – irony – nirol – ikomy’). Nonverbal intelligence was measured by the ‘Number-Connection-Test’ (Oswald and Roth 1997), a culture-free intelligence test that measures cognitive performance and processing speed and yields a nonverbal IQ.

#### Mood disturbance

Mood disturbance was obtained by the ‘Zerrsen Mood Scale’ (Von Zerssen 1975). The test consists of 28 pairs of antonymous words (e.g. happy-sad, satisfied-dissatisfied, serious-cheerful, energetic-weak, lethargic-active). The subjects have to decide which word of each pair most closely describes their current mood. Higher scores are correlated with dysphoria and depression (Heilman et al. 1975).

#### Aggressiveness

Information about aggressiveness was measured by the ‘Questionnaire for the Measurement of Aggressiveness Factors’ (Hampel and Selg 1975). This questionnaire includes 77 items related to hostility (e.g. ‘there is often bad blood between me and others’) and yields the factors *aggressiveness directed against others*, *self*-*directed aggression* and *inhibition of aggression*.

### Procedure

All participants were tested individually following the same procedure. The order of the materials was fixed with the assessment of mood disturbance and aggression in the first place, the assessment of nonverbal and verbal intelligence in the second place, and the cartoon-test in the last place.

### Data analysis

Factor analysis was performed in order to reduce the scores of the black humour cartoons to distinctive factors with an eigenvalue >1 (‘Kaiser-Guttman criterion’) using varimax rotation, followed by reliability analysis of the found factors, correlation analysis between age and the found factors as well as univariate differences with respect to gender and education level considering the black humour factors. A cluster analysis was calculated so as to find distinctive subject groups with respect to the black humour factors: comprehension, preference and vulgarity; verbal intelligence; nonverbal intelligence; mood disturbance; and aggressiveness factors: aggressiveness directed against others, self-directed aggression and inhibition of aggression. Discriminant analysis was calculated so as to analyse multivariate group differences. Univariate group differences were analysed for significance by analysis of variance, Kruskal–Wallis test, *T* test, *χ* ^2^-test and Gart’s 2I-test, depending on whether the expected frequency is lower than five in at least one class. Correlational analyses were conducted with respect to black humour factors: comprehension, preference and vulgarity; verbal intelligence; nonverbal intelligence; mood disturbance as well as the aggressiveness factors: aggressiveness directed against others, self-directed aggression and inhibition of aggression. The cut-off level for statistical significance was set at *p* < .05, 2-tailed. Data handling and analyses were carried out using SPSS for Windows, version 16.0.

## Results

Factor analysis with respect to black humour processing revealed three factors which explained 82% of the total variance. The first factor ‘comprehension’ explained 39% of the variance and consisted of the rating variables difficulty (loading of .94) and fit (.89). The second factor ‘preference’ accounted for 27% of the total variance and consisted of the four rating variables surprise (.76), novelty (.91), interest (.83) and preference (.77). The third factor ‘vulgarity’, only contained the variable vulgarity (.95) and explained 16% of the variance. Reliability analyses with respect to the black humour ratings yielded a Cronbach’s alpha of .78 for ‘comprehension’, .86 for ‘preference’ and .82 for ‘vulgarity’.

Correlation analyses yielded no significant relationships between age and black humour factors: comprehension (*r*(156) = −.112, *p* = .164), preference (*r*(156) = −.051, *p* = .530) and vulgarity (*r*(156) = −.078, *p* = .333). No significant differences with respect to gender were found regarding black humour factors: comprehension (*t*(154) = −1.8, *p* = .079), preference (*t*(154) = −.4, *p* = .686) and vulgarity (*t*(154) = .3, *p* = .757). No significant differences with respect to education levels were found regarding black humour factors: comprehension (*F*(3,152) = .3, *p* = .855), preference (*F*(3, 152) = .916, *p* = .435) and vulgarity (*F*(3, 152) = .919, *p* = .433).

Cluster analysis with respect to black humour factors: comprehension, preference and vulgarity; verbal intelligence; nonverbal intelligence; mood disturbance; and aggressiveness factors: aggressiveness directed against others, self-directed aggression and inhibition of aggression yielded three distinctive groups of subjects (for details see Table 2). The following discriminant analysis showed significant differences between the three groups (first function: canonical correlation = .8, Wilks’s Lambda = .2, *χ* ^2^(18, *N* = 156) = 255.9, *p* ≤ .0001; second function: canonical correlation = .7; Wilks’s Lambda = .5; *χ* ^2^(8, *N* = 156) = 103.5, *p* ≤ .0001). Ninety-eight per cent of the first group, 86% of the second and 95% of the third group were classified correctly, yielding an overall correct classification of 93% by black humour factors: comprehension, preference and vulgarity; verbal intelligence; nonverbal intelligence; mood disturbance; and aggressiveness factors: aggressiveness directed against others, self-directed aggression and inhibition of aggression. The highest correlations between the discriminant variables and the first standardized canonical discriminant function were found with respect to nonverbal intelligence (*r*(156) = .8) as well as between the discriminant variables and the second standardized canonical discriminant function with respect to mood disturbance (*r*(156) = .8).

**Table 2:** Socio-demographic variables; black humour comprehension, preference and vulgarity scores; verbal and nonverbal intelligence scores, mood disturbance; and aggressiveness with respect to the three humour groups

Variables	Group I (*n* = 41)	Group II (*n* = 50)	Group III (*n* = 65)
Age (years)	33.1 (11.7)	33.1 (12.7)	33.9 (11.7)
Females (frequencies)	20 (49%)	25 (50%)	31 (48%)
Males (frequencies)	21 (51%)	25 (50%)	34 (52%)
Black humour—comprehension*	2.8 (0.5)	2.8 (0.5)	3.1 (0.3)
Black humour—preference*	2.4 (0.4)	2.0 (0.4)	3.0 (0.4)
Black humour—vulgarity	2.3 (0.5)	2.3 (0.6)	2.3 (0.6)
Verbal intelligence*	101.0 (10.6)	96.8 (15.0)	109.7 (7.6)
Nonverbal intelligence*	97.8 (10.7)	102.8 (9.9)	118.1 (10.1)
Mood disturbance*^a^	9.2 (5.8)	19.5 (11.5)	8.6 (6.0)
*Aggression*			
Aggressiveness against others*^a^	11.2 (7.0)	14.5 (8.4)	9.4 (5.7)
Self-directed aggression^a^	3.5 (2.8)	4.6 (2.9)	3.6 (2.7)
Inhibition of aggression^a^	5.4 (1.7)	5.5 (1.9)	5.4 (2.3)

Scores are presented in means (standard deviations) and frequencies (percentage in respective group)

*Variables for which significant results could be found

^a^Higher scores represent more disadvantageous values

In the course of the discriminant analysis, univariate differences between the groups were analysed. Significant differences were found between the three groups with respect to nonverbal intelligence (*F*(2, 153) = 112.6, *p* ≤ .0001); verbal intelligence (*F*(2,153) = 19.9, *p* ≤ .0001); black humour factors comprehension (*F*(2,153) = 7.6, *p* = .001) and preference (*F*(2,153) = 5.5, *p* = .005); mood disturbance (*F*(2,153) = 46.9, *p* ≤ .0001); as well as aggressiveness against others (*F*(2,153) = 9.6, *p* ≤ .0001). With respect to black humour processing, group three shows the highest values with respect to comprehension and preference, whilst group two shows the lowest values regarding preference and group one and two do not differ with respect to comprehension. Taking a closer look at the intelligence scores, group three shows the highest values for both measures, whilst group two shows the lowest values regarding verbal intelligence and group one shows the lowest values regarding nonverbal intelligence. With respect to mood disturbance as well as aggressiveness against others, group two shows the highest values, whilst group three shows the lowest values. No univariate differences were found with respect to self-directed aggression (*F*(2,153) = 2.8, *p* = .066); inhibition of aggression (*F*(2,153) = .1, *p* = .921); black humour factor vulgarity (*F*(2,153) = 1.1, *p* = .342). See Table 2 for a detailed description.

The groups can be described as follows: the first group of subjects impresses with moderate black humour comprehension and preference, average nonverbal and verbal intelligence, low values in mood disturbances and moderate values in aggressiveness. The second group of subjects features moderate black humour comprehension and low black humour preference, average nonverbal and verbal intelligence, high values in mood disturbances and high values in aggressiveness. The third group of subjects shows high black humour comprehension and preference, high nonverbal and verbal intelligence, no mood disturbances and low values in aggressiveness (for details see Table 2). No significant differences between the three clusters were found with respect to age (*F*(2,153) = .097, *p* = .908) or gender (*χ* ^2^(2, *N* = 156) = .06, *p* = .970) (see Table 2), but with respect to education level (Gart’s 2I-test (6, *N* = 156) = 15.9, *p* = .014) showing more higher educated subjects in group III.

Correlational analyses yielded significant correlations between black humour comprehension and mood disturbance (*r*(156) = −.200, *p* = .012), nonverbal intelligence (*r*(156) = .177, *p* = .027), aggressiveness against others (*r*(156) = −.171, *p* = .033) as well as verbal intelligence (*r*(156) = .158, *p* = .049). Non-significant correlations were found between black humour comprehension and self-directed aggression (*r*(156) = .086, *p* = .284) as well as inhibition of aggression (*r*(156) = −.074, *p* = .361). Furthermore, significant correlations were shown between black humour preference and mood disturbance (*r*(156) = −.193, *p* = .016) as well as self-directed aggression (*r*(156) = .160, *p* = .045). Non-significant correlations were found between black humour preference and aggressiveness against others (*r*(156) = −.131, *p* = .102), inhibition of aggression (*r*(156) = −.052, *p* = .519), nonverbal intelligence (*r*(156) = −.035, *p* = .664) as well as verbal intelligence (*r*(156) = −.028, *p* = .729). Furthermore, significant correlations were shown between black humour factor vulgarity and aggressiveness against others (*r*(156) = .223, *p* = .005), mood disturbance (*r*(156) = .196, *p* = .014) as well as inhibition of aggression (*r*(156) = .179, *p* = .026). Non-significant correlations were found between black humour factor vulgarity and verbal intelligence (*r*(156) = −.113, *p* = .159), nonverbal intelligence (*r*(156) = −.088, *p* = .277) as well as self-directed aggression (*r*(156) = .022, *p* = .787).

## Discussion

The aim of this study was to investigate the cognitive as well as the emotional demands of black humour processing. The results of the current study show three distinctive groups with respect to comprehension as well as preference of black humour. The most surprising result is that subjects who show the highest values with respect to black humour preference and comprehension show high values with respect to intelligence, have higher education levels and show lowest values regarding mood disturbance and aggression. On the other hand, subjects who show average verbal and nonverbal intelligence scores as well as high mood disturbance and high aggressiveness show the lowest values with respect to comprehension and preference of black humour. These findings support the notion that humour processing depends on cognitive as well as emotional aspects (e.g. Ruch and Ekman 2001; Vrticka et al. 2013) and suggest that this also accounts for black humour processing which seems to be a complex information-processing task.

Previous studies suggested that aggressive mood leads to the preference of aggressive humour (e.g. Bowker and Etkin 2014; McCauley et al. 1983; Prerost 1983). Therefore, it would not have been a surprise for this study to show that subjects who enjoy reading cartoons dealing with nasty or morbid contents also show high levels of aggression. Quite surprisingly, it could be shown that subjects who present high levels of aggressiveness (i.e. aggressiveness directed against others) are most likely to dislike black humour and show lower values with respect to black humour comprehension than subjects with low aggression values. It can be hypothesized that higher levels of aggressiveness and associated levels of arousal lead to decreased levels of pleasure when reading black humour wits, an assumption which contradicts the results of Prerost (1983). Furthermore, it could be shown that subjects who are in a bad mood are most likely to dislike black humour and show lower values with respect to black humour comprehension than subjects who show low mood disturbance. These results support studies which show that subjective humour response is influenced by pre-existing mood (Neumann et al. 2001) as well as the notion that bad mood impairs the involvement in humour rather than facilitating the appreciation of aggressive humour (Ruch and Köhler 1998). According to Mindess and colleagues (1985), preference for sick humour is related to the ability to treat nasty contents as playful fiction. Seemingly, only those subjects who have no aggressive feelings towards others as well as no mood disturbance such as dysphoric or depressive mood can afford or get away with the playful exposure in the course of black humour processing. Another hypothesis would be that aggressiveness as well as bad mood could lead to a reduced information-processing capacity with respect to cognitively demanding humorous contents.

In this study, a strong association between black humour processing and verbal as well as nonverbal intellectual capacities can be shown. Subjects who show higher verbal and nonverbal intelligence scores show highest values with respect to black humour preference and comprehension. These results are in line with other studies which show a strong association between intelligence scores and humour processing (e.g. Greengross and Miller 2011) and indicate that such associations can also be seen for black humour processing. The role of intelligence in humour processing was recently investigated by Vrticka and colleagues (2013) in the light of the incongruity-resolution model (Suls 1972). They could show that in childhood and adolescence higher intelligence supports the detection of incongruities in a verbal utterance as well as the successful reinterpretation of these incongruities so as to get the joke. Furthermore, they could show that higher intelligence in this age-span is associated with stronger activity in brain areas involved in humour processing. Given the results of the current study, it can be hypothesized that in adulthood intelligence still strongly influences this two-stage problem-solving process with respect to humour processing. In this study, it could also be shown that the subjects who were most likely to comprehend and prefer black humour also have higher education levels. This result would be in line with another Freudian theory (1905) which states that subjects with high socio-economic status are more appreciative of aggressive humour (as long as it is thought to be within the bounds of good taste). This theory was not supported by McCauley and colleagues (1983), whilst the results of the current study showed that subjects with higher education show higher values with respect to black humour preference which is surprising considering the sinister, aggressive and tragic contents of black humour.

In the current study, a strong association between black humour comprehension and preference for black humour is shown. This result supports the findings of previous studies (Herzog and Bush 1994; Herzog and Karafa 1998) and indicates that higher comprehension of black humour wits leads to higher pleasure when reading it. Vrticka and colleagues (2013) indicate that intelligence not only influences the cognitive aspects of humour processing but also the affective components. In this context, the results of the current study as well as previous results (Coulson 2001) indicate that cognitive abilities like verbal and nonverbal intelligence as well as processing speed but also emotional aspects influence the mental operations underlying humour processing. On the one hand, it can be hypothesized that in the course of humour processing intelligence as well as mood and aggression directly influence the capacity to semantically reanalyse and reorganize categories and structures of thought (‘frame-shifting model’ by Coulson 2000), to combine these aspects into a meaningful humorous hybrid situation (‘frame blends model’ by Hofstadter and Gabora 1989; ‘blending model’ by Coulson 2001) and to adequately reinterpret the product of the previous mental operations (‘incongruity-resolution model’ by Suls 1972) so as to get the joke. It can be assumed that the extent to which each of these operations is needed for the comprehension of a joke varies depending on the content and structure of the joke. In this context, it can be hypothesized that intelligence, processing speed, aggression and mood not only influence the execution of the mental operations themselves but also facilitate the adapting of humour processing strategies in a quick and flexible way. The results in the current study support this hypothesis. On the other hand, it is likely that in the course of humour processing individual values with respect to intelligence, aggression and mood are likely to interact with the content of a joke. In the light of the previously mentioned models of humour processing, future studies could investigate whether mainly cognitive, mainly emotional or mixed contents require more mental effort.

## Conclusions

In the current study, three distinctive groups with respect to black humour processing can be shown. The most surprising result is that subjects who show the highest values with respect to black humour preference and comprehension show the highest values with respect to intelligence, have higher education levels and show the lowest values regarding mood disturbance and aggression. On the other hand, subjects who show average verbal and nonverbal intelligence scores as well as high mood disturbance and high aggressiveness show the lowest values with respect to comprehension and preference of black humour. Whilst a positive association between black humour processing and intelligence can be shown, aggressiveness and bad mood apparently lead to decreased levels of pleasure when dealing with black humour. Black humour processing is seemingly a complex information-processing task that depends on cognitive and emotional aspects. It can be hypothesized that these cognitive and emotional demands directly influence the mental operations underlying humour processing as they lead to an increased or decreased information-processing capacity but also facilitate the adapting of humour processing strategies in a quick and flexible way as humour processing is dependent on the content and structure of a joke.
